# Immunophenotyping of hemocytes from infected *Galleria mellonella* larvae as an innovative tool for immune profiling, infection studies and drug screening

**DOI:** 10.1038/s41598-024-51316-z

**Published:** 2024-01-08

**Authors:** Marialucia Gallorini, Beatrice Marinacci, Benedetta Pellegrini, Amelia Cataldi, Maria Luisa Dindo, Simone Carradori, Rossella Grande

**Affiliations:** 1 Department of Pharmacy, “G. d’ Annunzio” University of Chieti-Pescara, 66100 Chieti, Italy; 2 Department of Innovative Technologies in Medicine & Dentistry, “G. d’Annunzio” University of Chieti-Pescara, 66100 Chieti, Italy; 3 UdA TechLab, “G. d’ Annunzio” University of Chieti-Pescara, 66100 Chieti, Italy; 4 Department of Agricultural and Food Sciences, University of Bologna, 40127 Bologna, Italy

**Keywords:** Immunology, Adaptive immunity, Applied immunology, Infection, Inflammation, Innate immunity, Biological techniques, Animal disease models, Applied microbiology

## Abstract

In recent years, there has been a considerable increasing interest in the use of the greater wax moth *Galleria mellonella* as an animal model. In vivo pharmacological tests, concerning the efficacy and the toxicity of novel compounds are typically performed in mammalian models. However, the use of the latter is costly, laborious and requires ethical approval. In this context, *G. mellonella* larvae can be considered a valid option due to their greater ease of use and the absence of ethical rules. Furthermore, it has been demonstrated that the immune system of these invertebrates has similarity with the one of mammals, thus guaranteeing the reliability of this in vivo model, mainly in the microbiological field. To better develop the full potential of this model, we present a novel approach to characterize the hemocyte population from *G. mellonella* larvae and to highlight the immuno modulation upon infection and treatments. Our approach is based on the detection in isolated hemocytes from *G. mellonella* hemolymph of cell membrane markers typically expressed by human immune cells upon inflammation and infection, for instance CD14, CD44, CD80, CD163 and CD200. This method highlights the analogies between *G. mellonella* larvae and humans. Furthermore, we provide an innovative tool to perform pre-clinical evaluations of the efficacy of antimicrobial compounds in vivo to further proceed with clinical trials and support drug discovery campaigns.

## Introduction

The greater wax moth, *Galleria mellonella,* belongs to the Pyralidae family of the order Lepidoptera. The life cycle of this insect proceeds through four stages: egg, larvae (of different ages), pupa and adult. *G. mellonella* lives in beehives and larvae feed on honeycombs^1^. Research studies using in vivo models from invertebrates are becoming increasingly popular for investigations regarding the immune system because of their structural and functional similarity to the resistance displayed by mammals^2^. *G. mellonella* larvae are widely used as a model of infection for assessing the effectiveness of novel antimicrobial compounds before undergoing pre-clinical studies in mammals. Overall, *G. mellonella* has several characteristics which make it particularly suitable for a large-scale rearing, including adaptability to laboratory conditions and ease of handling. Moreover, costs are significantly lower compared to other models^3^. The rearing of *G. mellonella* may be performed according to standardized procedures and by varying several artificial diets, most of which partially based on ingredients contained in the larval natural food, i.e., honey and bee wax^4^.

The hemolymph of *G. mellonella* larvae consists of at least six types of hemocytes (plasmatocytes, granular cells, pro-hemocytes, coagulocytes, spherulocytes and oenocytoids), of which plasmatocytes and granular cells possess macrophage-like functionalities, being involved in phagocytosis and encapsulation^5^. However, the detailed interactions of hemocytes with pathogens is poorly understood and is complicated by the presence of different sub-populations of cells.

Several approaches based on the use of *G. mellonella* larvae are currently available and could be summarized into two main areas of study: the kinetics of survival after the testing of an infection-treatment and the host–pathogen interaction. Available techniques to evaluate immunomodulators or characterize hemocyte-pathogen interaction and the different cell types vary from the hemocytometer count, colorimetric assays, RT-PCR, 2D electrophoresis, fluorescent microscopy, reactive oxygen species or cytokine measurements by ELISA, detection of Annexin V or other apoptosis assays by flow cytometry and many others^6^.

The implementation of molecular biology is necessary to obtain more detailed information about the immune profile of hemocytes to disclose all the above-mentioned aspects of the study of *G. mellonella*^7^. To date, a protocol for an immunophenotypic analysis of the hemocyte population is not available. This study has been designed to develop an innovative approach for the characterization of *G. mellonella*-derived hemocytes and their immune activation. With this aim, cells from the hemolymph of *Staphylococcus aureus-*infected larvae with or without Vancomycin were isolated and analyzed by flow cytometry. Cell membrane markers typically expressed on monocytes/macrophages upon inflammation/infection (CD14, CD44, CD200), M1-polarization (CD80) and M2-polarization (CD163) were analyzed in the hemocyte population isolated. The panel of CD markers  set up for the analysis of hemocytes was used for the characterization of human monocytes and macrophages as comparison.

## Results

### Infection of *G. mellonella* larvae with *S. aureus*

**Figure 1:**
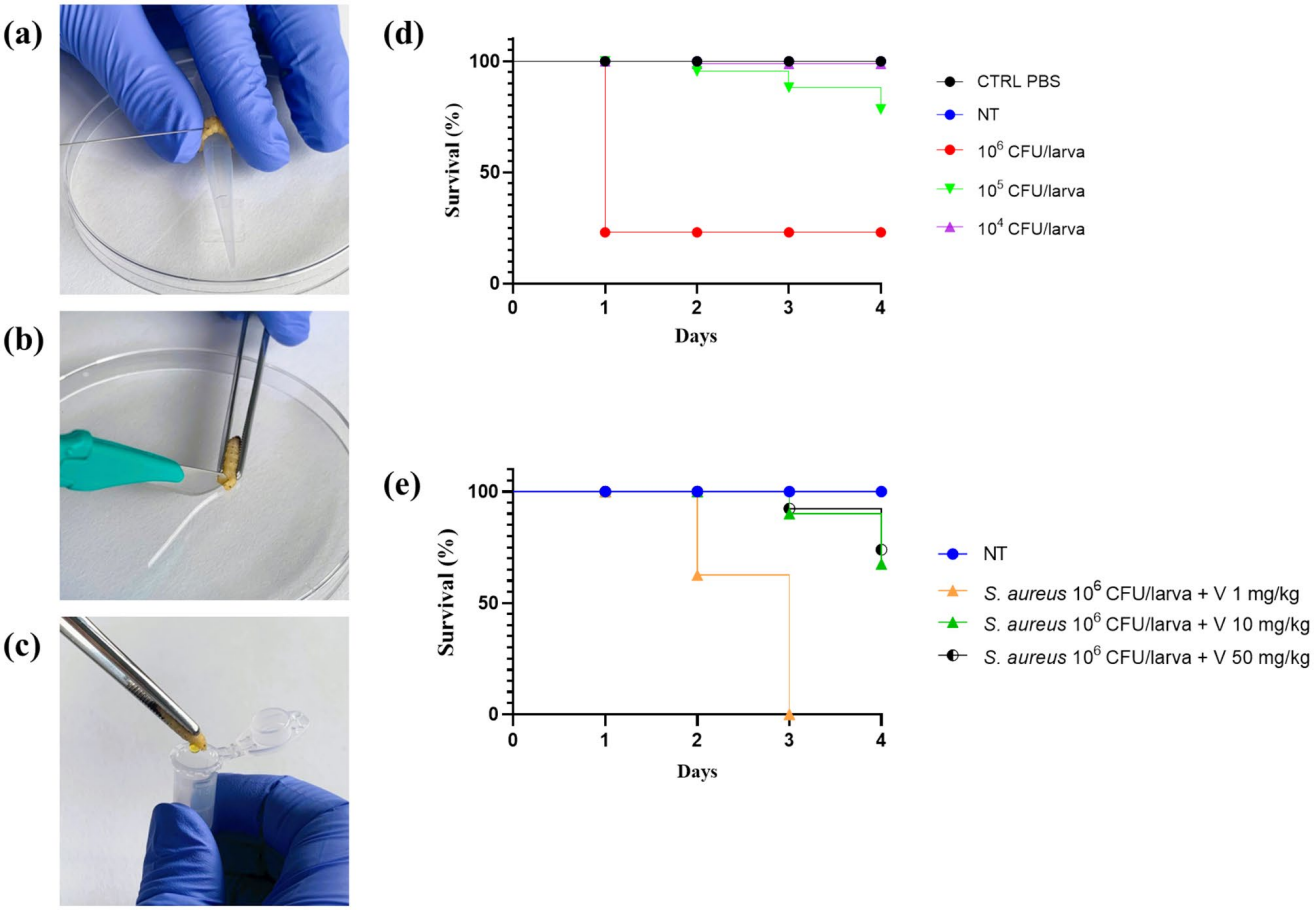
*G. mellonella* larvae manipulation, evaluation of the optimal *S. aureus* inoculum and Vancomycin dose. (**a**) Injection procedure (infection and Vancomycin treatment). (**b**) Incision of the larva with the sculp. (**c**) Hemolymph collection. (**d**) Effect of varying inoculum dose of *S. aureus* ATCC 43,300 on *G. mellonella* larvae survival. (**e**) Effect of treatment of *G. mellonella* larvae (inoculated with 10^6^ CFU/larva of *S. aureus* ATCC 43300) with Vancomycin (1, 10 and 50 mg/kg) (Kaplan–Meier survival curve).

At 24 h post infection, the highest inoculum dose tested results in a survival rate of 23% (Fig. 1d), therefore 10^6^ CFU/larva was selected for our experiments to mimic the worst scenario of infection. Vancomycin is effective on larval survival in a dose-dependent manner (Fig. 1e). Given these results, 50 mg/kg dose was selected for further experiments to obtain the best conditions of recovery.

### Cell count

**Figure 2:**
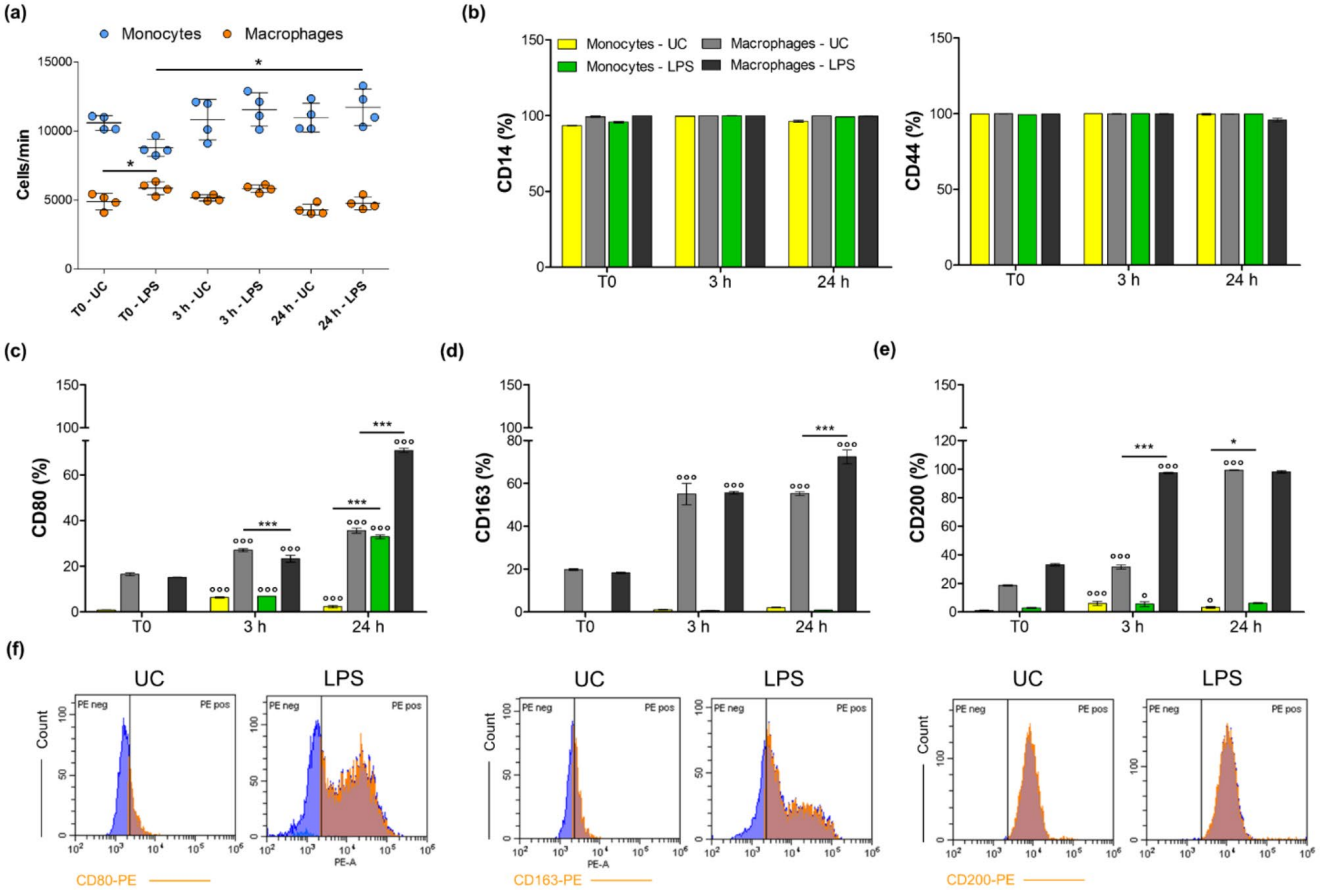
Cell count and immunophenotypic profile of human undifferentiated monocytes and macrophages at T0 and after 3 and 24 h from the LPS-stimulation. UC = untreated cells; LPS = cells stimulated by 0.5 µg/mL of LPS. (**a**) The dot graph displays the cell count expressed as cells/minute (*p < 0.01 between samples marked by lines). (**b**) Bar graphs show percentages of cells stained positive for anti-human mouse monoclonal CD14-FITC and CD44-FITC. (**c**, **d**, **e**) Bar graphs show percentages of cells stained positive for anti-human mouse monoclonal CD80-PE, CD163-PE and CD200-PE. *p < 0.01 and ***p < 0.0001 between samples marked by lines. °*p* < 0.01 and °°°*p* < 0.0001 between samples in the same experimental condition at different exposure times (T0 vs. 3 h and 3 h vs. 24 h). (**f**) Peaks of fluorescence emission (PE = phycoerythrin) generated by flow cytometry related to CD80, CD163 and CD200 expression in macrophages after 24 h of treatments (*x*-axis: cell count; *y*-axis: PE emission).

**Figure 3:**
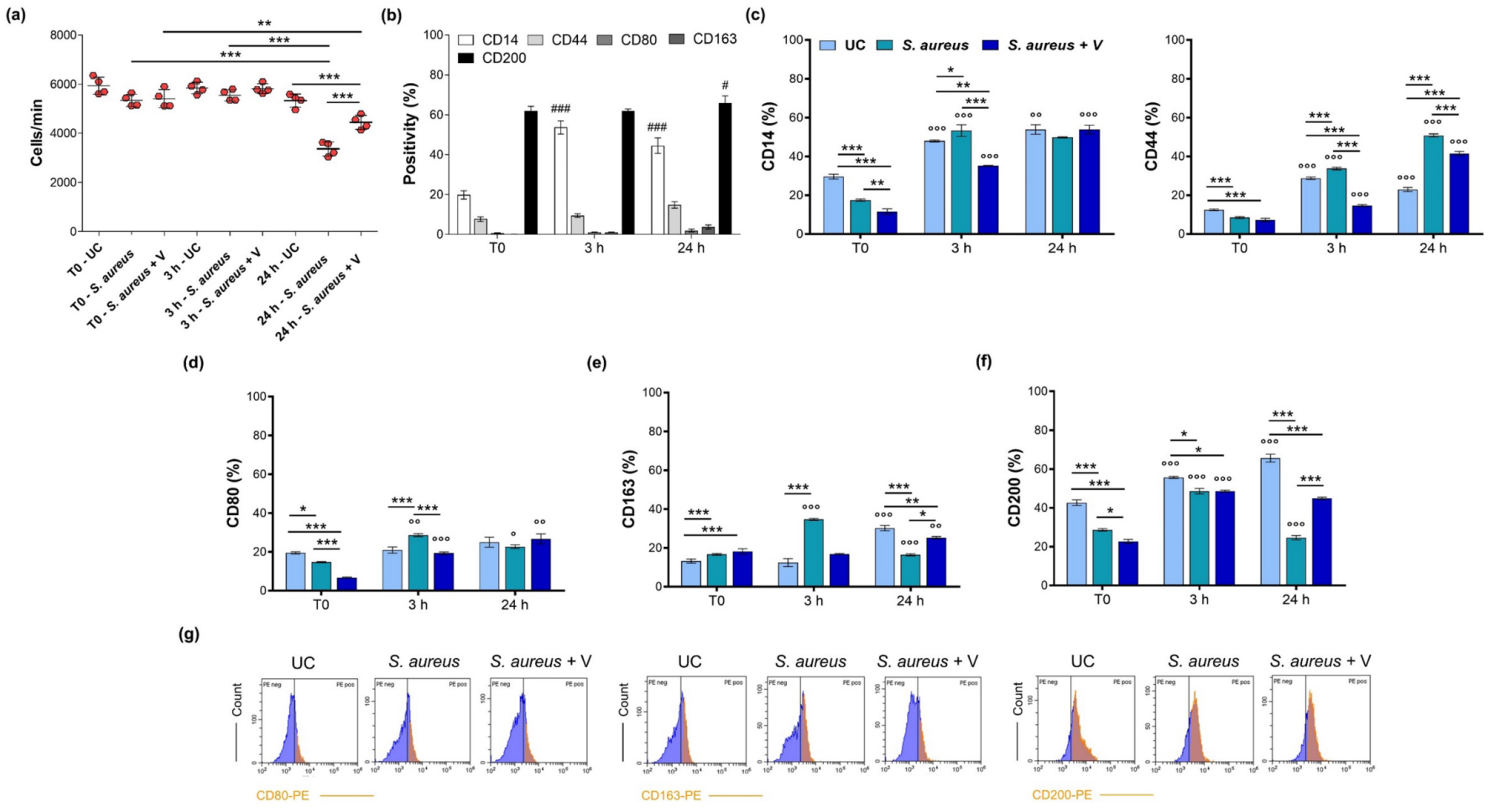
Cell count and immunophenotypic profile of *G. mellonella*-derived hemocytes at T0 and after 3 and 24 h from treatments. UC = untreated hemocytes; *S. aureus* = hemocytes isolated from *S. aureus*-infected larvae, 10^6^ CFU/larva; *S. aureus* + V = hemocytes isolated from larvae infected with *S. aureus* and treated with 50 mg/kg Vancomycin. (**a**) The dot graph displays the cell count expressed as cells/minute (**p < 0.001 and ***p < 0.0001 between samples marked by lines). (**b**) The bar graph shows percentages of LPS-stimulated cells stained positive for CD14-FITC, CD44-FITC, CD80-PE, CD163-PE and CD200-PE. #*p* < 0.01 and ###*p* < 0.0001 between markers at 3 and 24 h versus T0. (**c**) Bar graphs show percentages of cells stained positive for anti-human mouse monoclonal CD14-FITC and CD44-FITC. (**d, e, f**) Bar graphs show percentages of cells stained positive for CD80, CD163 and CD200. *p < 0.01 **p < 0.001 and ***p < 0.0001 between samples marked by lines. °*p* < 0.01, °°*p* < 0.001 and °°°*p* < 0.0001 between samples in the same experimental condition at different exposure times (T0 vs. T3 and 3 h vs. 24 h). (**f**) Peaks of fluorescence emission (PE = phycoerythrin) generated by flow cytometry related to CD80, CD163 and CD200 expression in hemocytes after 3 h of treatments (*x*-axis: cell count; *y*-axis: PE emission).

The number of undifferentiated monocytes is not affected by treatments except for cells exposed to LPS after 24 h which are weakly but significantly increased compared to cells at T0 (Fig. 2a). Likewise, no fluctuations are registered in the macrophage population other than between untreated and LPS-stimulated cells at T0. In parallel, the number of hemocytes was measured in the established experimental conditions (Fig. 3a). Although the number of cells is not influenced by treatments at T0 and after 3 h, exposures after 24 h are remarkably effective. The amount of hemocytes isolated from *S. aureus*-infected larvae is significantly lower after 24 h compared to all the experimental conditions at T0 and 3 h whereas the number of cells significantly increase in samples isolated from Vancomycin-exposed larvae.

### Expression of CD markers in human undifferentiated monocytes and macrophages

About 99% of both monocytes and macrophages express CD14 and CD44 in all the experimental conditions (Fig. 2b). CD80, a marker related to the macrophage M1 polarization is instead differentially expressed in both the cell populations and between treatments. While it is almost not expressed in the untreated monocyte population, a time-dependent increase in the percentage of cells CD80-positive is registered in LPS-stimulated cells (6.9% at 3 h and 32.9% at 24 h) (Fig. 2c). In parallel, the increase in CD80 positivity is time-dependent for untreated macrophages and this phenomenon is even more amplified for LPS-stimulated macrophages (15.9% at T0), being present a peak after 24 h of exposure (70.7%). Monocytes do not or only weakly express CD163, a marker related to M2 macrophages (Fig. 2d). Contrariwise, CD163 is upregulated in the macrophage population and the expression augments up to 24 h. In details, a remarkable increase in the positive population is registered after 3 h (about 55%) compared to T0 (about 20%), independently from LPS. After 24 h, the 72.4% of LPS-stimulated macrophages express CD163 while this percentage is comparable to the one registered after 3 h in the untreated control. Again, CD200 is only weakly expressed in monocytes at all the experimental times, being assessed around 5.5% (Fig. 2e). On the other hand, percentages of CD200-positive macrophages increase over the time of the experimental procedure, in a higher extend in LPS-stimulated samples. More specifically, the positivity varies from 33.3% (LPS, T0) to almost 99% after 24 h.

### Expression of CD markers in *G. mellonella*-derived hemocytes

**Figure 4:**
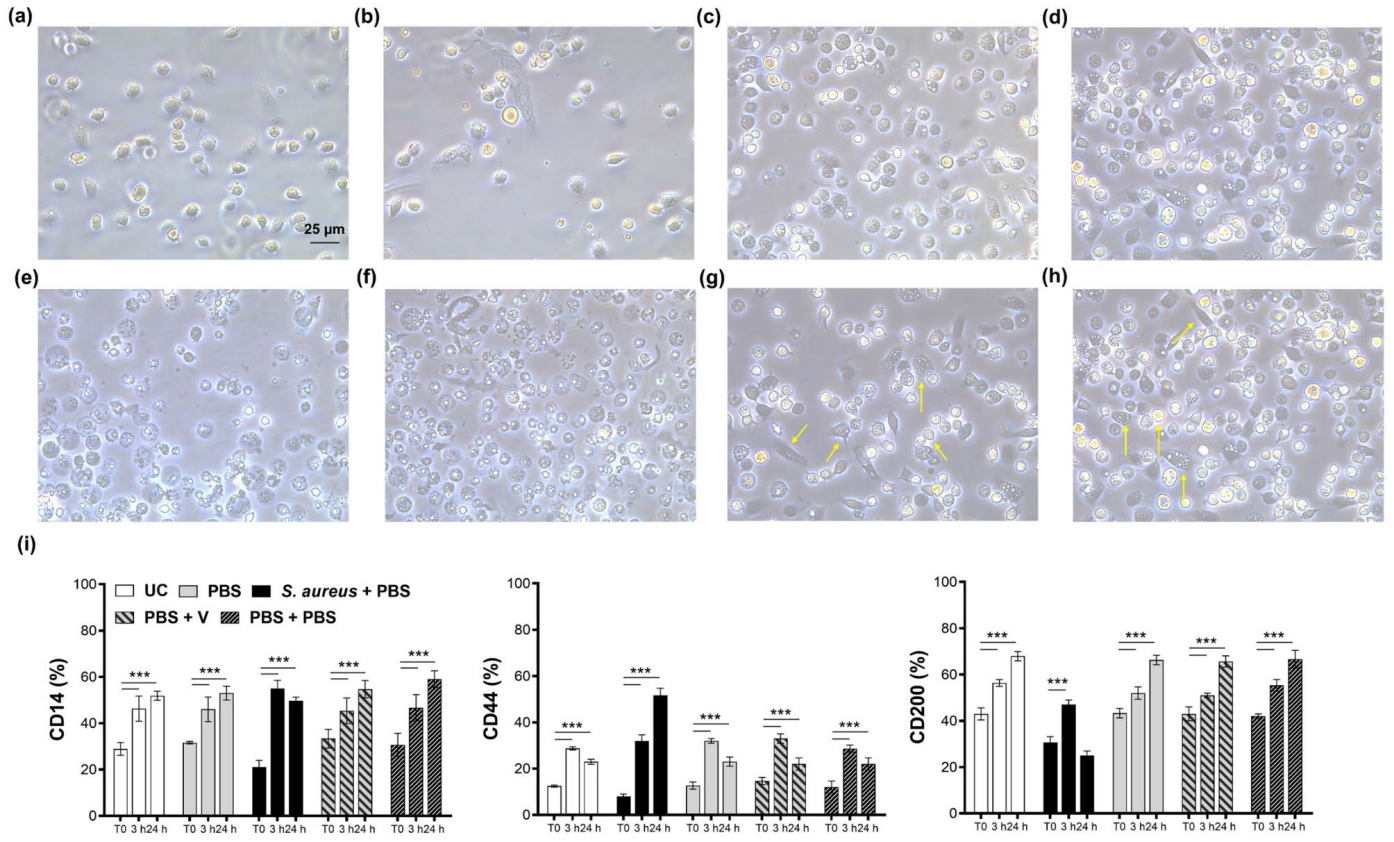
(**a-h**) Microscopic images of hemocytes. (**a**, **c**, **e**) Cells not resuspended in FACS buffer and (**b**, **d**,** f**) cells incubated in FACS buffer after 30 min, 3 and 24 h from seeding. (**g**, **h**) Images of hemocytes after 3 h from seeding. Differences in cell morphology in the same cell population are highlighted by yellow arrows. Magnification 40x. (**i**) Bar graphs show percentages of control groups for hemocytes stained positive for anti-human mouse monoclonal CD14-FITC, CD44-FITC and CD200-PE. ****p < 0.0001 between samples marked by lines. untreated larvae = UC; PBS injected larvae = PBS; *S. aureus* 10^6^ CFU/larva + PBS (30 min apart); PBS + Vancomycin (V) 50 mg/kg (30 min apart); PBS + PBS (30 min apart).

Hemocytes resuspended in FACS buffer present no signs of cell death and preserve their morphology compared to cells not resuspended in the buffer (Fig. 4a–f). Differences between the two experimental conditions are not remarkable after 30 min, 3 h and 24 h after isolation from larvae. Notably, differences in cell morphology within the whole cell population isolated from larvae is appreciable (Figure 4g, h). In parallel, the expression of CD14, CD44 and CD200 were measured in control groups (Fig. 4i), namely (I) untreated larvae (UC); (II) PBS-injected larvae (PBS); (III) *S. aureus* 10^6^ CFU/larva + PBS (30 min apart); (IV) PBS + V 50 mg/kg (30 min apart); (V) PBS + PBS (30 min apart). Levels of the same CD marker in between experimental conditions of control groups at the same experimental exposure appear to be very similar to the ones registered when larvae are treated with *S. aureus* and *S. aureus* + V (Fig. 3). It is thus plausible to assume that a double injection can alter the immune system of hemocytes over the time of the experiment but not as significantly if compared to further treatments.

To note, cells between the same isolated population display different cell morphology making plausible to assume that different type of hemocytes are present as reported elsewhere^5^.

The immunophenotypic profile of hemocytes isolated from LPS-stimulated *G. mellonella* larvae (LPS-hem) is shown in Fig. 3b. CD14 positivity is enhanced after 3 and 24 h, whereas CD44 is only weakly expressed. Next, CD80 and CD163 expression is only hardly detectable. Notably, these hemocytes display a significant positivity for the CD200 marker, independently from the time of exposure.

The positivity for CD14 in the hemocytes isolated from non-treated larvae (UC-hem) increases over the time of the experimental procedure, starting from 29.5% at T0 up to 53.8% after 24 h (Fig. 3c). In parallel, hemocytes derived from *S. aureus*-infected larvae (*Sa*-hem) disclose a similar pattern of positivity, although percentages are significantly higher than in UC. As for hemocytes isolated from larvae infected with *S. aureus* and subsequently treated with Vancomycin (*Sa*V-hem), the CD14 positivity is found decrease after 3 h compared to *Sa*-hem (35.1% and 53.2%, respectively). Percentages are comparable after 24 h in all the experimental conditions. About 10% of the hemocyte cell fraction is positive for CD44 at T0 (Fig. 3c). A dramatic increase is registered in the *Sa*-hem at 3 h (33.7%) and even more at 24 h (50.8%) compared to T0. In parallel, the presence of Vancomycin down-regulates CD44 expression (14.7% at 3 h and 41.4% at 24 h).

Next, about 20% of the whole UC-hem population is registered positive for CD80 in all the experimental conditions (Fig. 3d and 4g). The absolute higher percentage is registered when *G. mellonella* larvae are infected by *S. aureus* after 3 h (28.6%). Values are found comparable after 24 h of exposure. A similar trend is assessed for CD163 cell membrane expression at 3 h (Fig. 3e, g). In parallel, the CD marker is halved after 24 h in the *Sa*-hem population (16.4%) compared to UC-hem (30.1%) and weakly expressed in the presence of V (25.6%). Finally, CD200 positive cells are considerably high in the untreated population and percentages increase over the time of the experiment (42.6% at T0, 55.6% at 3h and 65.6% after 24 h) (Fig. 3f, g). On the other hand, CD200 percentages in *Sa*-hem disclose a less proportional time-dependent trend, being significantly increased after 3 h (48.5%) with respect to T0 (28.6%), but equally decreased after 24 h (24.6%). CD200 positivity percentages in this population are maintained significantly lower compared to UC in all the experimental times. In the presence of V, there is a similar trend of positivity at T0 and 3 h, but CD200 positive cells are higher after 24 h (44.9%).

## Discussion

*G. mellonella* larvae are a widely interesting invertebrate animal model for infectious disease research and, more recently, toxicology. As invertebrates, they own natural advantages over mice and rats for ethical, handling, and cost reasons. However, *Galleria* has serious limitations. It lacks many of the complex organ systems found in mammals, and like all other invertebrates, larvae do not have an adaptive immune system, although the innate system has proven to have similarities to that of humans^8^. Despite *G. mellonella* has not antigen-specific memory-based adaptive immunity, there is emerging evidence that larval immune responses are greatly enhanced in response to reinfections. Moreover, this immunological “memory” is epigenetically inherited by subsequent generations of insects^9^. In addition, recent studies have reported that immune priming is more similar to the phenomenon of “trained immunity” of vertebrate cells than to adaptive immunity *per se*^10^.

There is great interest in developing immunological methods that require fewer mammalian or non-mammalian donors. Thus, the use of insects as research models urgently requires the development of methods for working with hemocytes.

Cells of the human immune system are called leukocytes (or white blood cells). The leukocytes of innate immunity are classified as granulocytes (neutrophils, basophils, and eosinophils), macrophages, mast cells, dendritic cells (DCs), and natural killer (NK) cells. Each subpopulation owns its specific immunophenotypic profile, a process used to identify cells based on the types of antigens or CD markers on their surface^11^, typically run out by flow cytometry. In parallel, six types of hemocytes have been identified in the hemolymph of *G. mellonella*: plasmatocytes and granular cells, which are mainly involved in phagocytosis; pro-hemocytes, which may be stem cells able to differentiate into other hemocyte types; coagulocytes, participating in the hemolymph coagulation; spherulocytes, which mediate the secretion of cuticular components, and oenocytoids, involved in melanization^3,5,12^. Many studies of insect cells employ flow cytometry. A flow cytometric analysis has been used to characterize silkworm hemocytes from *Bombyx mori* using a fluorescent lectin staining^13^. Furthermore, a novel protocol has been established by Wrońska and co-workers^2^ for the intracellular cytokine detection based on flow cytometry in hemocytes from *G. mellonella* larvae. To the best of our knowledge, a study presenting a flow cytometric approach to analyze the immunophenotype of hemocytes in terms of expression of antigens on their membrane has not been reported, due to the lack of specific antibodies design for this usage.

Considering the reported shared characteristics between the human innate immune system and the one of *G. mellonella*, and that granular cells possessing macrophage-like functionalities have been identified in the hemolymph of G. *mellonella*^5^, an infection model of *G. mellonella* has been established with the aim to perform immunophenotypic analyses in vitro using anti-human antibodies towards macrophage CD markers modulated upon inflammation/infection.

*S. aureus* is an opportunistic pathogen responsible for nosocomial infections and a plethora of diseases ranging from skin infections to pneumonia, osteomyelitis and sepsis^14^. The treatment of such conditions is difficult due to the development of antimicrobial resistance versus drugs commonly used in therapies. In particular, Methicillin-Resistant *S. aureus* (MRSA) is associated with severe infections with increased mortality and morbidity^15,16^. The infection with *S. aureus* MRSA thus mimics an in vivo human sepsis, subsequently treated with Vancomycin to which the strain is susceptible, according to the MIC value. This study aims at evaluating the modulation of *G. mellonella* immune system over time and at comparing the results with the ones obtained in vitro by stimulating human undifferentiated monocytes and macrophages with LPS. For comparison, larvae were stimulated also with LPS, to mimic the infection from Gram negative bacteria. The data obtained confirmed a similarity between the in vitro and in vivo models, suggesting that the characterization of the immune system of *G. mellonella* could represent a suitable pre-clinical model to validate the anti-inflammatory and the immunomodulatory properties of natural and synthetic compounds.

CD14 is a lipopolysaccharide-binding protein, functioning as an endotoxin receptor. It is found strongly expressed in monocytes and most tissue macrophages but monoblasts and promonocytes are weakly positive or negative for this marker. Myeloblasts and other granulocytic precursors do not express CD14, but neutrophils and a small proportion of B lymphocytes may weakly express it^17^. In parallel, adhesive interactions between CD44 and hyaluronan (HA) have been implicated in the regulation of immune cell trafficking within various tissues. More in details, it has been found that CD44 is involved in the leukocyte recruitment cascade upon inflammation and infection (rolling, firm adhesion, trans-endothelial migration and chemotaxis)^18^. In our experimental model, undifferentiated monocytes, and macrophages strongly express CD14 and CD44, independently from the LPS presence, as expected. On the other hand, CD14 and CD44 are differentially expressed in the hemocyte population upon the experimental conditions, disclosing a more heterogeneous distribution of cell population among the whole cell fraction analyzed. In details, the increase of CD14 and CD44 positivity in the infected hemocyte population at 3 h and 24 h, is paralleled by a decrease when larvae are exposed to Vancomycin. Data disclose that CD14 and CD44 are weakly expressed by hemocytes under basal conditions and thus their expression is inducible and modulated by infection. CD14^+^ cells are considered to be mostly macrophages and monocytes, although some studies indicate that neutrophils express CD14 at low levels^19^. Additionally, evidence suggests that CD44 is a physiological human neutrophil E-selectin ligand^20^. This observation lay the grounds for further in-depth analysis to decipher the modulation of CD14 and CD44 on the hemocyte membrane, suggesting the investigation of markers more related to the neutrophil population.

According to their inflammatory status, macrophages are classified as classically activated (pro-inflammatory, M1), non–activated (M0) and alternatively activated (anti-inflammatory or pro-resolving, M2) cells, a classification also associated with distinct genetic profiles and expression of specific surface markers. CD163 is a macrophage specific scavenger receptor for haptoglobin-hemoglobin complexes found on the cell membranes of M2 macrophages. Its expression is strongly induced by the anti-inflammatory cytokine IL-10, making CD163 a marker of the anti-inflammatory process occurrence. On the contrary, CD80^+^ cells are classically M1 macrophages^21^. As expected, CD80 and CD163 are expressed on human macrophages but not in undifferentiated monocytes, and their expression is amplified by the presence of LPS. In the hemocyte population, CD80 cell positivity is weak and seems weakly influenced by the presence of the infection induced by *S. aureus*. In parallel, there is an increase in the CD163 cell positivity in *Sa*-hem after 3 h but levels are comparable among the experimental conditions after 24 h. This observation reinforces the precedent one, which is that a macrophage-like population is present in the hemocyte population but might disclose different markers onset compared to the human one.

Finally, expression of CD200 was analyzed. CD200 is a transmembrane protein related to the B7 family of co-stimulatory receptors involved in T-cell signaling and likely plays a role in physiologic immune tolerance. It is normally expressed on lymphoid and neuronal tissues, and its receptor, CD200R, is found on antigen-presenting cells and T-cells. Additionally, the classical macrophage activation is inhibited by the CD200 receptor (CD200R) and the CD200/CD200R immune-checkpoint pathway has been widely demonstrated to maintain immune homeostasis during infection by preventing excessive activation of macrophages^22^. Our data confirm that CD200 mediates the inflammatory response in the macrophage population, being the percentage of positive cells increased in the presence of LPS. In the hemocyte population, CD200 increases in the untreated population over the time of the experiment and decreases in infected cells, and then increases again after 24 h with Vancomycin (Fig. 3f). To survive in a host, many bacterial and parasitic pathogens adopt the CD200-CD200R axis by modulating the expression of either CD200 or CD200R1, which in turn attenuates the innate immunity. For example, *Leishmania amazonensis* induces the expression of CD200 both at mRNA and protein levels in bone marrow macrophages, which next inhibit neighboring macrophages expressing CD200R1, and thus, abrogating nitric oxide (NO) production during the infection^23^. It has been reported that CD200 significantly suppresses the *S. aureus*-induced production of NO and pro-inflammatory cytokines in mouse macrophage^24^. The decreased expression of CD200 upon *S. aureus* infection suggests therefore a more in-depth analysis of this pathway in the hemocyte population.

Our novel approach confirms that invertebrates and vertebrates share evolutionary conserved components among innate immune responses^3,25^ as hemocytes from *G. mellonella* larvae react with anti-human antibodies commonly used for immunophenotyping in vitro. In parallel, our analysis discloses that the hemocyte population is highly heterogeneous, being the immunophenotypic profile of hemocytes significantly different from the one of a homogeneous monocytic/macrophagic cell line. Therefore, a wider panel of CD markers related to the whole leukocyte population might be established to discriminate the various population subtype and to deeply understand the molecular mechanism underlying the hemocyte activation. Finally, it has been demonstrated that hemocytes from *G. mellonella* are highly responsive to infections/inflammation insults and their immunophenotype is modulated by drugs in parallel, equally to human blood monocytes. Although with limitations regarding the use of anti-human antibodies to discriminate hemocytes from an invertebrate, profiling the immunophenotype of *G. mellonella* larvae in vitro could be therefore a suitable tool for the screening of vaccines and new compounds and substances acting as immunomodulators or antibiotics.

## Methods

### Monocytes/macrophages (cell culture, in vitro differentiation and stimulation)

Undifferentiated human monocytes (CRL-9855™) were purchased from ATCC® and sub-cultured in RPMI 1640 (Merck, Darmstadt, Germany) supplemented with 10% heat-inactivated fetal bovine serum (FBS), 1% penicillin/streptomycin, and 1% sodium pyruvate (all from Gibco, Invitrogen, Life Technologies, Carlsbad, CA, USA) at 37 °C and 5% CO_2_. Differentiated macrophages were obtained as previously described^26^. Both the cell types were seeded at 0.5 × 10^5^ cells/well in multi-well culture plates (Corning, Falcon®, Glendale, Arizona, USA). To establish an inflamed environment, cells were stimulated with LPS 0.5 µg/mL (lipopolysaccharide from *Escherichia coli*, purchased from Merck, Darmstadt, Germany, stock solution 1 mg/mL in water). Untreated and LPS-stimulated samples were harvested immediately after treatment (T0 = time zero) and after 3 and 24 h and used for further procedures.

### *G. mellonella* larvae

*G. mellonella* larvae were obtained from the laboratory colony available at the Department of Agricultural and Food Science (DISTAL) of the University of Bologna (Italy) and stored in the dark at 37 °C until use.

At DISTAL, the colony was maintained at 30 ± 1 °C, 65 ± 5% RH, 0:24 L:D photoperiod, according to the methods described by Dindo and Francati^27^. The larvae were kept in plastic boxes (24 × 8 × 8 cm) and fed on the artificial diet developed by Campadelli^28^ composed of skimmed milk powder, white wheat flour, whole wheat flour, maize flour, brewer’s yeast, beeswax, wildflower honey and glycerin. To obtain eggs, about 100 cocooned mature larvae approaching pupation were placed in plastic boxes (3.5 L volume) with a 6-cm diameter hole on their lids. Holes were covered with filter paper discs which were fixed to the lids with adhesive tape. Adults emerged 5-6 days after pupation, mated and females laid eggs on the paper disc. Eggs were collected 2–3 times a week and placed in new boxes with diet, which was supplied every 2–3 days until the end of larval development, which proceeds through 6–7 instars and lasts approximately 30-35 days. Adult moths do not feed^29^. Sixth-instar larvae (about 2 cm long) were used for the experiments. 24h before the experiments, larvae were weighed, selected and kept in a separate box without diet.

### Infection of *G. mellonella* larvae

*S. aureus* ATCC 43300 was used in this study. The strain was cultured on Mueller Hinton Agar plates (MHA, Oxoid) at 37 °C in aerobic conditions. Bacteria were transferred in 8 mL of Mueller Hinton II Broth (MH2B; Sigma-Aldrich) and incubated at 37 °C, 125 rpm in aerobiosis. After 16 h of incubation, bacteria were harvested by centrifugation at 10.000 rpm for 5 min, 4 °C. The supernatant was discharged, the cellular pellet was washed in Phosphate Buffered Saline (PBS; Sigma Aldrich) followed by another step of centrifugation as before. Bacterial cells were re-suspended in PBS and the optical density was measured at 600 nm (OD_600_) to obtain the proper bacterial suspension. Larvae weighing within 200–250 mg were selected for the experiments. Each administration required the injection of a volume corresponding to 10 μL in the third left pro-leg of the larva (Fig. 2). Two injections were required, therefore, the second one was performed on the third right pro-leg of the larva.

To determine the optimal infection dose, groups of *G. mellonella* larvae (n = 10 per group) were injected with different suspension of *S. aureus* (10^6^-10^5^-10^4^ CFU/larva) and incubated in Petri dishes at 37 °C for 4 days to score mortality. Controls groups included: (I) untreated larvae; (II) PBS injected larvae. After establishing the proper inoculum dose, suitable for our experiments (10^6^ CFU/larva), the in vivo efficacy of different doses of Vancomycin (V) was assessed. At 30 min post-infection, larvae were randomized to receive 1 mg/kg, 10 mg/kg or 50 mg/kg^30^ of the antibiotic and then incubated at 37 °C in a Petri dish to score mortality. The Minimum Inhibitory Concentration (MIC) of Vancomycin for *S. aureus* ATCC 43300 corresponded to 1 μg/mL and was previously determined in vitro via the broth microdilution method^31,32^. After determining the proper bacterial inoculum (10^6^ CFU/larva) and the Vancomycin dose (50 mg/kg), *G. mellonella* larvae were divided into two groups of treatment, i.e. (I) *S. aureus* 10^6^ CFU/larva and (II) *S. aureus* 10^6^ CFU/larva + V 50 mg/kg. An additional group was injected with LPS from *E. coli*: (III) LPS 1 µg/larva. The controls groups included: (I) untreated larvae (UC); (II) PBS injected larvae; (III) *S. aureus* 10^6^ CFU/larva + PBS (30 min apart); (IV) PBS + V 50 mg/kg (30 min apart); (V) PBS + PBS (30 min apart).

### Hemolymph extraction

Hemolymph extraction was performed at different time points: (I) T0, immediately after injection; (II) 3 h post injection; (III) 24 hours post injection. Each larva was anesthetized on ice for 1–2 min before gently cut one of the last abdominal segments with a scalp (Figure 1a, b): the hemolymph was allowed to drain out and collected in a sterile tube (Figure 1c). After collecting the hemolymph from two larvae belonging to the same treatment group, 20 μL of the hemolymph pool were harvested with a micropipette, transferred in a sterile tube and mixed with 100 μL of an anticoagulant solution (93 mM NaCl, 100 mM glucose, 30 mM trisodium citrate, 26 mM citric acid, 10 mM Na_2_EDTA, and 0.1 mM phenylthiourea, pH 4.6), prepared as reported elsewhere^33^.

### Cell count and immunophenotyping of undifferentiated monocytes, in vitro differentiated macrophages and hemocytes isolated from *G. mellonella*

After the established exposure times (T0, 3 and 24 h), the number of undifferentiated monocytes, macrophages and hemocytes from *G. mellonella* was assessed by flow cytometry (CytoFLEX, Beckman Coulter, CA, USA).

Isolated hemocytes were incubated for 1 h in FACS buffer prepared with 10 mM 4-(2-hydroxyethyl)-1-piperazineethanesulfonic acid (HEPES) buffer at pH 7.4, 140 mM sodium chloride (NaCl), and 2.5 mM calcium chloride (CaCl_2_), and afterwards seeded on cell culture-treated 6 well plates (Corning Incorporated, NY, USA). Not incubated and FACS incubated cells were placed in RPMI 1640 (Merck, Darmstadt, Germany) supplemented with 10% heat-inactivated fetal bovine serum (FBS), 1% penicillin/streptomycin, and 1% sodium pyruvate (all from Gibco, Invitrogen, Life Technologies, Carlsbad, CA, USA) at 37 °C and 5% CO_2_. Cells were afterwards observed by means of a phase-contrast microscope equipped with a camera (Leica). Images were acquired and analyzed by the Leica Application Suite LAS EZ version 3.4 (Leica, Wetzlar, Germany) immediately after seeding, after 3 and 24 h.

**Figure 5:**
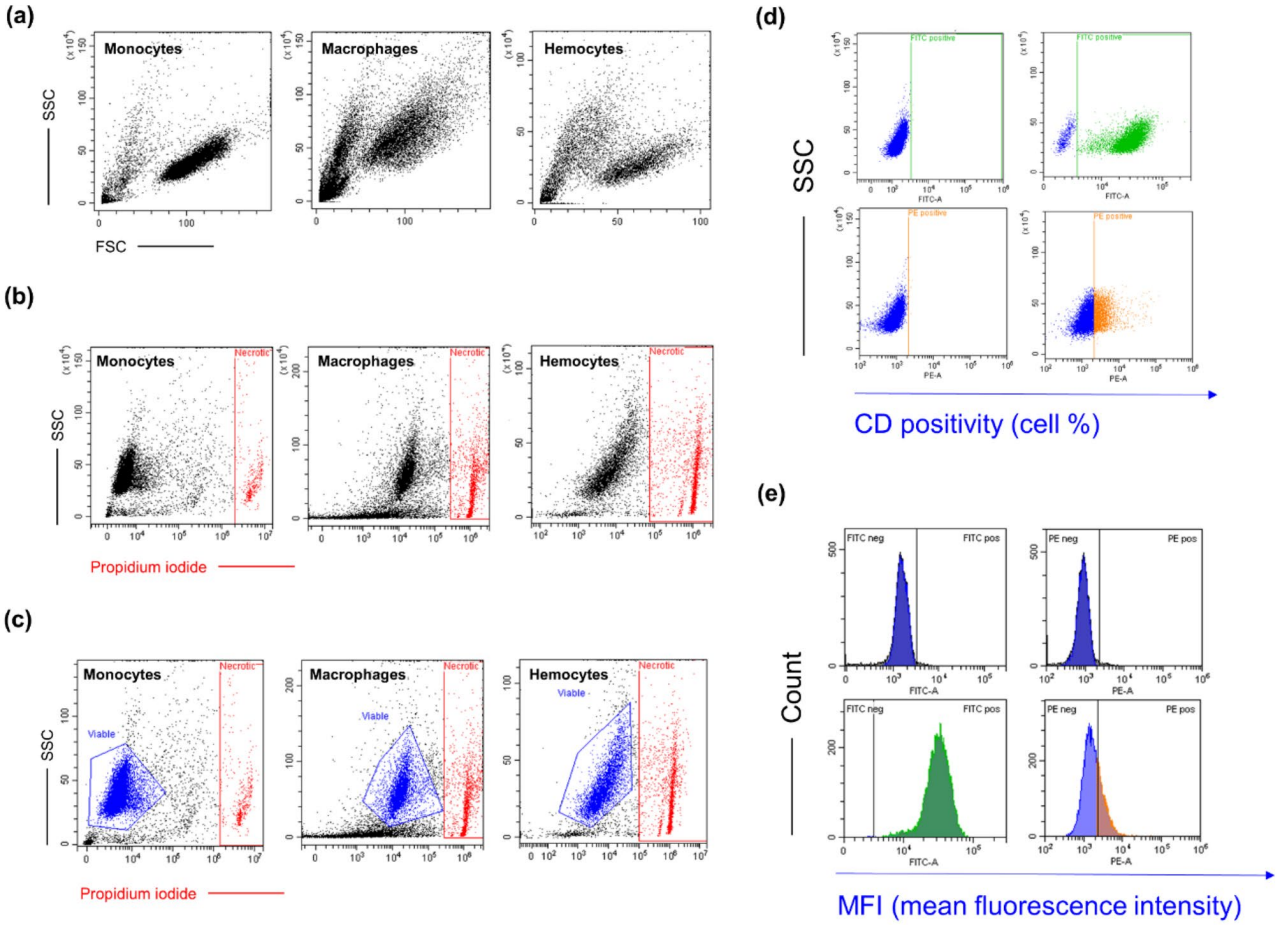
Gating strategy for the immunophenotype analysis performed by flow cytometry. (**a**) Side scatter/Forward scatter (SSC/FSC) dot plots represent morphological parameters of unstained monocytes, macrophages, and *G. mellonella*-derived hemocytes. (**b**) Each cell type was incubated with anti-human clusters of designation (CDs)—fluorochrome conjugated and propidium iodide in parallel to assess the necrotic cell population. Cells stained positive were gated and labeled as necrotic. (**c**) The cell population excluded from the logical gate “necrotic” (in blue) represents the viable population used for further analyses. (**d**) The SSC/FITC (fluorescein) or PE (phycoerythrin) dot plots represent cells incubated with antibody isotypes (FITC and PE-conjugated) used to set the fluorochrome threshold (negative control). Right-shifted cells in the FITC and PE gates were considered positive for the marker analyzed. Data were expressed as the percentage of positive cells. (**e**) Histograms show peaks of fluorescence emission in the FITC and PE channels. Right-shifted peaks are directly proportional to the number of positive cells for the marker analyzed and they are expressed as mean fluorescence intensity (MFI).

As for FACS analyses, before running samples, cells were stained by propidium iodide (PI). Briefly, PI 10 µg/mL (stock solution = 1 mg/mL) was added to each sample for 10 minutes. Next, cells were run at FACS and gated by their morphological parameters (Side Scatter/Forward Scatter, SSC/FSC) excluding the necrotic population (Fig. 5). Next, defined flow rate (medium) and acquisition time (1 min) were set up. Data were expressed as the number of cells in the morphological gate of viable cells refracting the laser emission within 1 minute through the CytExpert Software 5.0 (Beckman Coulter, CA, USA).

Next, the expression of surface markers (CDs) was analyzed using flow cytometry. After the exposure times, cells were harvested, collected by centrifugation in the cold, and washed once with FACS buffer Cells were incubated with fluorochrome-conjugated antibodies (1:50 dilutions) in 50 μL of FACS buffer for 15 min in the dark. Cells were stained separately in each single screening tube with a panel of anti-human mouse monoclonal antibodies: CD14-FITC, CD44-FITC, CD80-PE, CD163-PE and CD200-PE (all purchased by BD Biosciences, MA, USA). Then, the excess of antibodies was removed by adding fresh FACS buffer and by centrifugation. Before running 20,000 events (for monocytes and macrophages) and 10,000 events (for hemocytes) in a Beckman Coulter CytoFLEX flow cytometer (Brea, CA, USA), cells were incubated with propidium iodide (PI) to exclude necrotic PI-positive cells from the analysis (Figure 5). Relative fluorescence emissions of gated cells by forward and side scatter properties (FSC/SSC) were analyzed using the CytExpert Software (Beckman Coulter) and results were expressed as the percentage of positive cells for each CD marker. Individual values obtained from independent experiments (n = 6) were summarized as means and standard deviations.

## Data Availability

The datasets used and/or analysed during the current study available from the corresponding author on reasonable request.
